# Examining Share plus—A Continuous Glucose Monitoring Plus Data-Sharing Intervention in Older Adults and Their Care Partners: Protocol for a Randomized Control Study

**DOI:** 10.2196/60004

**Published:** 2024-12-16

**Authors:** Nancy A Allen, Cynthia A Berg, Eli Iacob, Bruno Rodriguez Gonzales, Jonathan E Butner, Michelle L Litchman

**Affiliations:** 1 College of Nursing University of Utah Salt Lake City, UT United States; 2 Department of Psychology University of Utah Salt Lake City, UT United States

**Keywords:** type 1 diabetes, T1D, older adults, continuous glucose monitoring, data sharing, dyadic coping, diabetes management, diabetes self-care, glucose monitoring, quality of life, mobile phone

## Abstract

**Background:**

Older adults with type 1 diabetes (T1D) are increasingly turning to care partners (CPs) as resources to support their diabetes management. With the rise in diabetes technologies, such as continuous glucose monitoring (CGM), there is great potential for CGM data sharing to increase CP involvement in a way that improves persons with diabetes’ glucose management and reduces distress.

**Objective:**

The specific aims of this paper are to (1) evaluate the feasibility, usability, and acceptability of the Share plus intervention compared to the CGM Follow app plus diabetes self-management education and support; (2) evaluate the effect of the Share plus intervention on time-in-range (TIR; primary outcome) and diabetes distress (secondary outcome); and (3) explore differences between groups in person with diabetes and CP dyadic appraisal and coping, quality of life, diabetes self-care, and CP burden at 12 and 24 weeks and associations of dyadic variables on outcomes.

**Methods:**

This is a protocol for a feasibility, pilot randomized controlled trial. Older adults with T1D and their CP (N=80 dyads) will be randomized 1:1 to the Share plus intervention or Follow app plus diabetes self-management education. The trial will include a 12-week active intervention to determine the change in primary (TIR) and secondary (diabetes distress) outcomes, followed by a 12-week, observation-only phase to examine maintenance effects. The evaluation is guided by the Dyadic Coping Model. Patient-level effectiveness outcomes (TIR, hemoglobin A_1c_ [HbA_1c_], diabetes distress, diabetes appraisal, coping, quality of life, diabetes self-care behaviors, and CP burden) will be assessed, using patient-reported outcomes measures and a home HbA_1c_ test kit. Patient- and CP-level acceptability and feasibility will be assessed using surveys and interviews. Quantitative feasibility, acceptability, and usability data will be described using frequencies and percentages. Acceptability will be summarized based on Likert questions and open-ended questions. Usability will be examined separately for the intervention and control groups based on the System Usability Scale, with a study benchmark of ≥68 indicating good usability. TIR will be computed based on 2 weeks’ worth of data at baseline (prior to intervention) and 2 weeks each after the intervention (week 12) and at follow-up (week 24).

**Results:**

Recruitment started in August 2023 and enrollment began in November 2023. To date, 24 participants have been enrolled in this study. We expect to conclude this study in March 2026 and expect to disseminate results in March 2026.

**Conclusions:**

To our knowledge, this will be the first pilot randomized controlled trial to evaluate both feasibility and effectiveness outcomes for the web-based, platform-delivered Share plus intervention for older adults with T1D and their CP. This research has implications for CGM data sharing in other age groups with T1D and type 2 diabetes.

**Trial Registration:**

ClinicalTrials.gov NCT05937321; https://clinicaltrials.gov/study/NCT05937321

**International Registered Report Identifier (IRRID):**

DERR1-10.2196/60004

## Introduction

### Background

Type 1 diabetes (T1D) is a significant public health problem, with increasing numbers of adults now living into late adulthood [1]. T1D self-management requires a number of daily diabetes tasks including checking blood glucose (either through a blood glucose meter or a continuous glucose monitor [CGM]) and administering insulin to account for changes in food intake and exercise. Older adults with T1D are at increased risk for hypoglycemia, hyperglycemia, and glucose variability that may result in seizures, falls, and myocardial infarctions [2,3]. These risks are due to a number of factors including reduced awareness of hypoglycemic warning symptoms; reduced hormonal counterregulatory response; and changes in dexterity, visual acuity, cognitive function, depression, and anxiety that may prevent affected individuals from taking corrective actions [3,4].

Care partners (CPs; eg, spouse, adult child, romantic partner, and friend) can serve as important resources for older adults living with T1D in reducing these risks to self-management [5-9]. Over half of the adults with diabetes have an unpaid CP who regularly assists with diabetes management [8], and age-related changes often increase persons with diabetes’ need for CP assistance. One tool that is available to persons with diabetes and their CPs to reduce harmful glucose levels is real-time CGM. A CGM transmits glucose trend data to the smartphone of persons with diabetes and provides predictive alarms before problematic and potentially dangerous hypoglycemia and hyperglycemia levels [10]. Recommendations for standards of care in diabetes [11] now include the use of CGM with older adults with T1D to address increased risks of hypoglycemia. Persons with diabetes are given glucose goals of 70-180 mg/dL, and the percentage of time spent within this range is called time-in-range (TIR), an important metric for evaluating glucose targets [12,13]. In older adults, CGM is effective at decreasing glucose variability, with older adults highly adherent to wearing CGMs [14,15]. The data provided by the CGM can now be shared with a CP’s smartphone to facilitate diabetes support for the person with diabetes [16]. Yet, few older adults and CPs are sharing data with CGMs [17].

There is great potential for CGM data sharing to increase CP involvement in a way that improves glucose targets and reduces distress for persons with diabetes. For instance, the free data-sharing app, Dexcom Follow [18], allows CGM readings to be displayed on the smartphone or smartwatch of persons with diabetes and their selected CPs. Follow also allows the user to see CGM glucose levels and receive predictive hypoglycemia and hyperglycemia alerts. Additional apps such as the free Dexcom Clarity app [19] use the data from CGM to create glucose data reports to allow persons with diabetes and their selected CPs to identify glucose patterns and trends and has also been associated with a greater quality of life (QoL) when reports are viewed with a CP [14]. However, few older adults share their CGM data or review clarity reports with a CP. Data from the Wireless Innovation for Seniors with Diabetes Mellitus trial showed that only 9.2% of older adults with T1D used data sharing, and only 32% used their smartphones to monitor their CGM data [15]. In our preliminary studies, older adults with diabetes reported an increased perceived need to have others monitor their glucose levels for safety purposes [20], but few persons with diabetes are adopting this technology.

This intervention was designed to maximize the use of data sharing by persons with diabetes and their CPs. Our prior work identified several barriers to using data sharing involving dyadic communication that involved patients’ and partners’ different expectations regarding family involvement [20]. Persons with diabetes frequently regarded diabetes as “their own illness,” whereas spouses viewed the illness as more shared [21,22]. Improving collaboration and communication among those with type 2 diabetes [23] was associated with lower distress in persons with diabetes and their partners, higher satisfaction, and improved glycemic levels (among those with moderately elevated hemoglobin A_1c_ [HbA_1c_]).

**Figure 1:**
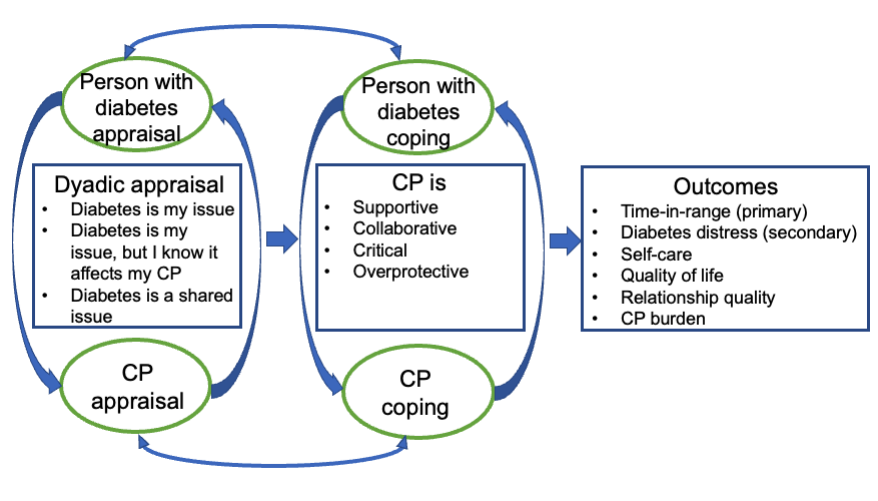
Dyadic coping process affected by Share plus intervention (reproduced from Bristol et al [27], which is published under the terms of Creative Commons Attribution 4.0 license [28]). CP: care partner.

The intervention is guided by the Dyadic Coping Model (Figure 1) [24,25], a widely used framework for how individuals with chronic illness and CPs can be involved in chronic illness management. As applied to diabetes, dyadic coping involves two components: appraisal of the illness and coping efforts. Appraisal refers to whether the persons with diabetes and their CPs perceive diabetes as “our” problem versus “my” or “your” problem. Shared appraisal can initiate and facilitate coping strategies that are more collaborative to address diabetes stressors that are also perceived as shared [26]. When a person with diabetes appraises diabetes as an illness that is shared with a CP, persons with diabetes report more collaborative involvement from their spouse, greater relationship satisfaction, and less regimen distress [22]. Challenges can arise when one person in a dyad (eg, the person with diabetes) appraises diabetes as their own issue to deal with and the CP engages in high levels of involvement (eg, wishes to be very involved in data sharing). That is, collaborative involvement of the CP may be detrimental when the person with diabetes views diabetes as only their illness to deal with and does not consider its effects on the CP. The Dyadic Coping Model supports the value of a CP’s collaborative involvement in a person with diabetes’ glucose monitoring via CGM.

The Share plus intervention framed by the Dyadic Coping Model begins with a discussion of how persons with diabetes and CPs appraise diabetes (eg, patients alone or shared with CP) and the next communication regarding the preferred ways of involving the CP in data sharing (Figure 1). For instance, the person with diabetes is asked what words would be helpful and supportive from their CPs in response to a high or low glucose alarm and what might be viewed as critical or overprotective. Next, CPs are asked if this communication is acceptable to them and if they are willing to provide this requested support. In our preliminary studies, persons with diabetes who were initially hesitant to share their glucose data reported high satisfaction at 12 weeks [29,30]. These discussions facilitate an understanding of diabetes as a shared illness and the collaborative and supportive strategies that the CP can engage in. Moreover, these discussions match data-sharing strategies that fit with a person with diabetes’ appraisal of diabetes and are viewed as supportive rather than unsupportive. Such discussions will facilitate an action plan that will best align with the appraisal process of the person with diabetes and their CP.

### Study Objectives

This study aims to (1) evaluate the feasibility, usability, and acceptability of the Share plus intervention compared to Follow plus diabetes self-management education (DSME); (2) evaluate the effect of the Share plus intervention on TIR (primary outcome) and diabetes distress (secondary outcome); and (3) explore differences between groups in person with diabetes and CP dyadic appraisal and coping, QoL, diabetes self-care, and CP burden at 12 and 24 weeks and associations of dyadic variables on outcomes.

## Methods

### Study Design

This study will use a 1:1 randomized controlled trial to conduct a pilot test of 80 dyads to compare the Share plus intervention to the Follow plus DSME intervention. The trial will include a 12-week active intervention to determine the change in primary (TIR) and secondary (diabetes distress) outcomes, followed by a 12-week, observation-only phase to examine maintenance effects. The SPIRIT (Standard Protocol Items: Recommendations for Interventional Trials) guidelines were used in reporting this study [31].

### Setting

This clinical trial will be conducted in Utah, and participants will be recruited at the state level and the national level using telehealth.

### Sample and Recruitment

Persons with diabetes and CPs are actively being recruited from 3 major health care systems in Utah in which we have identified champions who have assisted with recruitment in our preliminary studies. The University of Utah Enterprise Data Warehouse will be used to identify eligible persons with diabetes. An opt-out strategy will be used using letters, postcards, emails, and SMS text messages. Additionally, the Electronic Data Warehouse will be used to build a recruitment dashboard allowing the research team to reach out to contact high-volume providers to inform them of the study and to recruit participants in person before or after clinic visits.

Recruitment inside and outside of Utah will include (1) digital marketing strategies and (2) diabetes clinician and provider networks. Digital marketing strategies include posting to T1D social media groups. Professional groups, such as the Association of Diabetes Care and Education Specialists (ADCES), will also be contacted to post information and digital flyers about the study. We are committed to reaching racial or ethnic minorities at rates at least equivalent to their proportion in our catchment area population [32] as required by National Institutes of Health policy [33] and by our own strong commitment to principles of equity and social justice.

### Eligibility Criteria

Participants with diabetes will be eligible if they (1) are aged 60 years or older, (2) have a T1D diagnosis (may add more detail here), (3) are currently using CGM, (4) have HbA_1c_ ≥7.5 and ≤11%, (5) are able to read and write in either English or Spanish, (6) are able to manage diabetes with respect to insulin administration and glucose monitoring (which may include assistance from a CP), and (6) are naïve to using the Follow app and are willing to use the Follow app. Participants with or without an insulin pump are eligible. Participants will be excluded if they have (1) a life expectancy estimated at <1 year, (2) extreme visual or hearing impairment that would hinder the ability to use CGM, (3) stage 4 or 5 renal disease, (4) a history of psychiatric or psychosocial issues that could limit adherence to required study tasks, or (5) a Montreal Cognitive Assessment score <19 indicating moderate to severe dementia.

CP participants will be eligible if (1) the participant with diabetes identifies them, (2) they are aged 18 years or older, (3) they are able to read and write in either English or Spanish, and (4) they are willing to participate in a data-sharing telehealth intervention study with the participant with diabetes. CPs will be excluded if they have a self-reported diagnosis of moderate or severe dementia or other medical conditions that make it inappropriate or unsafe to fulfill the role of a CP. CPs will not be directly recruited.

### Group Randomization

Persons with diabetes (n=40) and CPs (n=40) who meet the study criteria and agree to participate will be asked to sign an informed consent with a digital signature. The persons with diabetes will be randomly assigned to 1 of 2 groups (40 dyads in the Share plus intervention group and 40 dyads in the Follow plus DSME group) using a computer-generated schedule of randomly permuted blocks of sizes 2, 4, and 6 to minimize knowledge of the next participant’s assignment. Group allocation will be executed in REDCap (Research Electronic Data Capture; Vanderbilt University).

### Description of Intervention and Education Groups

#### Share plus Intervention

##### Overview

The Share plus intervention will be delivered by Certified Diabetes Care and Education Specialists (CDCESs) over three 60-minute sessions using a Health Insurance Portability and Accountability Act (HIPAA)–compliant Zoom (Zoom Video Communications) meeting platform with audio transcription, with 2 additional dyad phone calls to reinforce the counseling sessions (Table 1). The 3 Share plus intervention sessions introduce new and increasingly advanced educational material and are designed to overcome the persons with diabetes and CPs’ reluctance in data sharing and to improve diabetes management. This includes training in dyadic CGM communication and problem-solving, leading to a data-sharing action plan.

**Table 1:** Intervention and education group activities.

Week	Intervention group—over the web with Dyad Follow + Share plus	Control group—over the web with Dyad Follow + DSME^a^

0	Baseline CGM^b^ data collection for 14 days; no active intervention Survey and home HbA_1c_^c^ kit completion Set up Clarity to allow access to data	Baseline CGM data collection for 14 days; no active intervention Survey and home HbA_1c_ kit completion Set up Clarity to allow access to data
2	Start Share plus intervention Set up the Follow app Start the Share plus intervention Communication and problem-solving strategies Detailed action plan including glucose targets	Start usual care Set up the Follow app Two education handouts provided covering ADCES7^d^ self-care education topics: Monitoring and Taking Medication
3	Dyadic phone call Reinforce the Share plus intervention and provide technology support	—^e^
4	Share plus intervention + glucose pattern management with focus on food choices and healthy eating Review communication and problems New communication content Revise action plan as needed for communication and problem-solving hypoglycemia and hyperglycemia Dyadic glucose pattern management training using Clarity, set automatic Clarity downloads Dyad set goals for regular times to discuss glucose trends and problem-solving,	Usual care intervention Review goals and progress Two education handouts provided on ADCES7 topics: Reducing Risks and Health Eating
6	Dyadic phone call Reinforce the Share plus intervention and provide technology support	—
8	Share plus Intervention + glucose pattern management with lifestyle (exercise, stress, illness, etc) Review communication and problems Dyadic glucose pattern management using Clarity Dyadic review of glucose targets after an in-depth analysis of CGM data and dyadic treatment strategies to improve glucose TIR^f^	Usual care interventions Review goals and progress 3 education handouts ADCES7 handouts on Being Active, Problem Solving, Healthy Coping
12	Data collection—end of active intervention Measures and home HbA_1c_ kit completion. Retrieve TIR data from Clarity	Data collection—end of active intervention Measures and home HbA_1c_ kit completion Retrieve TIR data from Clarity
24	Data collection—end of observation phase Measures, home HbA_1c_ kit completion, Clarity download	Data collection—end of observation phase Measures, home HbA_1c_ kit completion, Clarity download

^a^DSME: diabetes self-management education.

^b^CGM: continuous glucose monitoring.

^c^HbA_1c_: hemoglobin A_1c_.

^d^ADCES: Association of Diabetes Care and Education Specialists.

^e^Not applicable.

^f^TIR: time-in-range.

##### Step 1: Shared Appraisal Assessment

Dyads are asked how they appraise diabetes with the following question: “When you think about diabetes, is it: (1) my issue to deal with as an individual, (2) my issue but I know it affects my CP, or (3) a shared issue.” The CPs are asked the same question, but “my” is substituted with “his or her.” A follow-up question will be asked, “Help me to understand why you selected this response.” If a person with diabetes or CP views diabetes as their own, they are asked to consider something that was shared. The CDCES explores why it is shared and if it is easier to work on the problem together than alone. Next, persons with diabetes and CPs are each asked about their confidence in sharing glucose data. Using scripted motivational interviewing questions, persons with diabetes and their CPs are asked to discuss their answers. The objective of this discussion is to determine the initial comfort level with CGM data sharing within the dyad.

##### Step 2: Communication

Dyads are asked how comfortable they feel about data sharing. Examples are provided about how other persons with diabetes have described the benefits of sharing their diabetes, such as an increased sense of teamwork, support, QoL, and decreased diabetes-related burden. The barriers to sharing glucose levels are also identified (eg, glucose levels are private and the person with diabetes does not want to be judged). The person with diabetes next identifies effective and ineffective communication strategies around sharing hyperglycemia and hypoglycemia. The person with diabetes is asked (1) how they feel about their partner seeing their glucose levels and any concerns they have about sharing their glucose data; (2) how they would like their CP to respond to their glucose numbers, and specifically what words or actions are viewed as supportive and helpful and what is unhelpful, nagging, or controlling behavior; and (3) if or how they want their CP to help them to figure out the cause of a low or high glucose level. Education is provided about steps for clear communication. Finally, the CP is asked how they feel about this type of communication and if it is acceptable. The objective of this discussion is to explore supportive and unsupportive conversation strategies and foster their commitment to data sharing.

##### Step 3: Problem-Solving

The dyad is asked to work together to set alarms on the smartphones of the persons with diabetes and CPs (they can be set differently), set the volume of alerts (eg, can cause frustration), or use vibrate mode. First, CPs are asked to confirm their willingness to be a safety net for emergencies and look at alarms. The persons with diabetes are asked to acknowledge their willingness to look at their alarms. Next, dyads are coached to identify the expectations and length of waiting time before the CP should contact the person with diabetes for a concerning glucose level and problem-solve an agreeable strategy for different alarms. For example, when a CP gets a low alarm with two arrows indicating a more rapidly decreasing glucose level, how long should they wait to contact the person with diabetes; how long should they wait for reply; and if there is no reply, what action should they take? This discussion also includes the preferred mode by which the CP will contact the person with diabetes (eg, phone call, text, and email). The dyad is also asked to discuss glucose trends once per week and then problem-solve if they will do this, when, and how. The objective of this third step is to guide the dyad in making and agreeing upon boundaries around data sharing.

##### Step 4: Action Planning

Dyads are asked to agree in writing how, when, and if they would like to be contacted for specific alarms as outlined in step 3. The type of communication that is identified as supportive responses to hyperglycemia and hypoglycemia are recorded in the action plan from step 2. The action plan is documented and given to the dyad to set clear expectations around data sharing.

##### Step 5: Re-evaluating, Practicing, and Advancing

At weeks 4 and 8, dyads will review their experience using data sharing and discuss their concerns regarding communication, dyadic problem-solving around hypoglycemia and hyperglycemia, and alarms, so that they can update their plan. Supplemental information is provided (as needed) on communication strategies (eg, listening, reframing, being positive, and providing appreciative feedback). Dyads are asked to practice problem-solving skills related to their biggest identified problem in the previous weeks with data sharing. The Clarity app produces several reports, including an ambulatory glucose profile that helps to determine glucose trends and patterns that can be downloaded on a computer or smartphone. The CDCES will have data-driven conversations from these reports at each session, and through shared decision-making, will develop a plan to help increase TIR to 70-180 mg/dL. In session 2, the CDCES will instruct the dyad on how to use the Clarity app to download reports of glucose trends, how to review glucose patterns, and strategies to address hypoglycemia or hyperglycemia patterns with a focus on food choices and healthy eating. Dyads will be instructed on setting up weekly automatic Clarity downloads and asked to set aside time to discuss these reports. In session 3, the CDCES will review the Clarity download with a new focus on glucose pattern management with lifestyle changes (exercise, stress, sleep, and illness). The action plan will be revised as needed at each session.

Handouts are provided at baseline to the dyads that support topics in each Share plus session. The format of the handouts includes education on topics such as hypoglycemia, CGM-specific actions to take to treat hypoglycemia, and tips for CPs on how to be supportive when the person with diabetes is experiencing hypoglycemia.

#### Overview of Education Group—Follow plus DSME Intervention

Dyads in the Follow plus DSME Intervention will receive 3 diabetes education sessions (Table 1). The CDCES will cover topics from the ADCES7 educational curriculum including healthy coping, healthy eating, being active, taking medication, reducing risks, and problem-solving in 3 sessions. ADCES handouts are provided at baseline on the ADCES7 educational topics.

### Outcomes and Measures

Feasibility, usability, and acceptability measures will be collected throughout the study, both quantitatively and qualitatively. Feasibility for this study is operationalized to include the ability to recruit the target population, retention of participants, participant adherence to the study protocol, CDCES fidelity in delivering the intervention, and the intervention dose provided. Our team will measure intervention fidelity as a continuous process to ensure that intervention components are delivered in a standardized way through in-depth training and standardized protocols. This will include a 10% review of Zoom intervention and control sessions conducted by 2 CDCESs. Data will be collected and reviewed in an ongoing basis for attendance, adherence to the intervention script, delivery of assigned topics, and self-report of intervention delivery and completion per study protocols. The research team will use an observation form to collect intervention fidelity data on a 4-point scale (from 0=none to 3=high level of intervention fidelity) to monitor the intervention sessions. This observation form will be used to counsel CDCESs and to monitor for any intervention delivery problems. Usability includes measures of processes to complete the intervention and a measure used to examine the reliability and validity of the telehealth and Share plus technology intervention. Process measures include appointment attendance, length of all sessions, number of unscheduled appointments for extra assistance, and number of telephone calls for support by the persons with diabetes or CPs. Engagement data include persons with diabetes and CPs’ frequency of viewing glucose data and responding to alarms; intent to continue using Follow; and a tailored System Usability Scale, to assess reliability or validity of the telehealth and Share plus technology intervention and to determine usability between groups and against System Usability Scale benchmarks. Acceptability will include persons with diabetes and CPs’ satisfaction with the Share plus intervention. Acceptability will be assessed using a 7-item Likert scale and 4 open-ended questions developed in our preliminary studies.

The 3 Share plus sessions will be conducted on HIPAA-compliant Zoom and recorded and transcribed using the meeting transcription feature. Dyadic data will be collected qualitatively in our intervention feasibility data and quantitatively. The qualitative feasibility intervention data includes collaborative involvement, cooperative actions, challenges, supportive or critical communication, problem-solving discussions regarding hyperglycemia and hypoglycemia, solutions discussed then implemented, unsuccessful and successful solutions, and barriers to protocol completion. Quantitative measures usability measures include process measures such as appointment attendance, length of all sessions, number of unscheduled appointments for extra assistance, and number of telephone calls for support by the persons with diabetes and CPs. Engagement data include persons with diabetes and CP’s reported frequency of viewing glucose data, responding to alarms, and intent to continue using the Follow app. Other quantitative measures will be available at baseline and at 12 and 24 weeks, including interpersonal processes such as diabetes appraisal [22], social support [34], relationship quality [35], diabetes self-care [36], CP burden [37], QoL [38], and percentage of protocol completion. The primary outcomes are TIR and diabetes distress [39] and partner diabetes distress [40]. CGM data will be collected from the Clarity reports for 14 days at each time point (Clarity website stores data continuously). Other descriptive measures include glycemic metrics from CGM data [41].

### Data Management

All data will be entered into the REDCap database, with data storage on a secure, dedicated research server. CGM data will be collected from the Clarity reports for 14 days each at baseline, 3 months, and 6 months. All individuals involved in screening and data entry will be trained to use the database. The research team and the principal investigator will review entered data for completeness and accuracy.

### Ethical Considerations

The study procedures were approved by the University of Utah Institutional Review Board (NIDDK Share plus: Continuous Glucose Monitoring with Data Sharing in Older Adults with T1D and Their Care Partners, 00160673). Consistent with best practices in research, informed consent will be collected from the participants, and they have the ability to opt out at any time. All data will be deidentified to protect the privacy of participants. Participants will be compensated US $50 at baseline, 12 weeks, and 24 weeks.

### Statistical Methods and Analysis

#### Study Aim 1 Analysis

Quantitative feasibility, acceptability, and usability data will be described using frequencies and percentages. Acceptability will be summarized based on Likert questions and open-ended questions. Usability will be examined separately for the intervention and control groups based on the System Usability Scale [42], with a study benchmark of 80% or more of participants having a score of ≥68 indicating good usability. The qualitative data from intervention transcripts will be checked for transcription accuracy. Next, feasibility data will be qualitatively coded, line-by-line, using principles of qualitative thematic analysis [43,44]. Codes from a codebook will be compared, contrasted, and collapsed to develop corresponding themes [45-47].

#### Study Aim 2 Analysis

Our primary research question asks if the percentage of TIR (70-180 mg/dL) will be significantly higher in the Share plus versus the control group. TIR will be computed based on 2 weeks’ worth of data at baseline (prior to intervention) and 2 weeks each after the intervention (week 12) and at follow-up (week 24). Primary efficacy evaluation expectation would show no difference for percentage of TIR at baseline but improvement in the Share plus condition at 12 weeks in comparison to the control. Further, improvement at 24 weeks in comparison to control would be suggestive of a maintained benefit. We will assess the efficacy of our intervention for percentage of TIR using a multilevel model with a random intercept. Time will be captured through 2 dummy codes treating the baseline as the referent. The significance and direction of the first dummy code capture change from baseline to 12 weeks. The significance and direction of the second dummy code capture change from baseline to 24 weeks. Condition will then moderate these effects as a means to determine the efficacy. Follow-up analyses will compare 12 to 24 weeks if some efficacy is still found at 24 weeks in comparison with baseline. Exploratory analyses will include examining age, duration of T1D, education level, and number of complications as covariates. In all cases, mean differences along with 95% CIs will be reported. All tests will be evaluated using α=.05 (two-tailed).

#### Study Aim 3 Analysis

Our exploratory question asks what differences in person with diabetes and CP dyadic appraisal and coping, QoL, diabetes self-care, and CP burden will be found between the Share plus intervention versus the control group. We will explore differences between the groups on these measures, analogous to the multilevel models in study aim 2, separately for persons with diabetes and CPs. Exploratory evaluation will be performed through a between-by-within interaction with the expectation of there being no difference for measures at baseline but significant differences between the two conditions at 12 and 24 weeks. For the second half of the aim, we will explore questions like “Does improvement in self-care behaviors and dyadic relationship quality lead to improvements in persons with diabetes’ TIR?” In this case, we will use regression models with persons with diabetes’ TIR at 24 weeks regressed on baseline TIR and difference change score of self-care at 12 weeks minus baseline. Interaction terms with treatment will be included to look at the impact of the intervention on these associations. While we recognize that the study is underpowered for formal analysis, these preliminary trends can inform future fully powered dyadic and mediation analyses to determine the underlying processes that are influenced by the intervention.

#### Power and Sample Size

Power analyses were conducted in G*Power 3.1 (Heinrich-Heine-Universität Düsseldorf) based on population-level values. It will parallel the results of a Monte Carlo study as long as there is minimal missing data and assumptions (eg, normality) are met. We deemed these acceptable assumptions for the proposed study. Power calculations were based on the *F* statistic from within-by-between, mixed design, repeated-measures ANOVA. We stipulated an n=80, power=0.80, and α=.05 with 3 repeated-measures time points and 2 between-level groups. This parallels aims 2 and 3, examining for an intervention by time interaction such that no difference is expected at baseline, but a difference should be detectable at 12 and 24 weeks. The sample size allows us to detect an *F* of 0.15, which corresponds to a *d*=0.3, which falls into the small to moderate effect size range following Cohen’s suggested benchmarks. This would be sufficient given that we wished to detect a minimally important difference of ≥5% improvement in TIR, which with an SD of 20% corresponds to Cohen *d* of 0.25. Detecting a smaller effect size even with a larger sample size would not have as much clinical utility. Analyses and power calculations are similar for the diabetes distress scale. With a clinically meaningful change of 0.5 points and an SD of 1.5-2.0, this corresponds to *d*=0.25 to *d*=0.3. Therefore, the power to detect *d*=0.3 as described above for TIR will also be sufficient to detect a meaningful change in the distress scale. For both sample size calculations, there is minimal loss in effect size detection with losing as much as 20% of the expected sample size. This implies that we should be fairly robust to power loss due to attrition, technical issues, or other reasons for expecting incomplete data, though data loss should be fairly minimal for TIR, with the common occurrence being technical issues that might lead to 2-3 of the days lost for calculating % out of range. Of note, given that we will use *Best Linear IMPutation* to impute missing data, this, therefore, provides a conservative estimate of power and sample size.

#### Missing Data

The proposed analyses will be based on all available observations in combination with multiple imputations to avoid distorting variance or inducing bias [48]. We operationalize adherence as the mean hours that CGM is worn per day. The CGM r package *iglu* [49,50] uses linear interpolation for customizable small windows of missing data (eg, 20 min) within the hours specified. For longer periods of missing data beyond 20 minutes, the data will not be interpolated by iglu as this may bias results, based on current recommendations for CGM [51]. Instead, we will use the *Best Linear IMPutation* program, which can generate a fully conditional multiple imputation solution for multilevel data structures [51]. The missing-completely-at-random test will be used to identify necessary imputation predictors. This methodology should be efficient for both missing at random and missing completely at random circumstances. Missing data patterns will be examined and discussed by the team experts to consider if any may be suggestive of nonrandom missingness. Similar procedures will be applied to the validated behavioral measures.

#### Data Monitoring

Our team has developed a study operations manual and an intervention manual building on the manuals developed in our feasibility studies [29,30,52]. These manuals are used to ensure maximum compliance and protection with good clinical practices for research and include processes such as recruitment, informed consent, and protection of personal health information and video data. Participants complete electronic surveys using a web link to REDCap, a HIPAA-compliant, web-based application hosted at the University of Utah Center for Clinical and Translational Science Institute, which securely stores and protects data. No names appear on any surveys and will only be used by the study team. The qualitative data collected from the Zoom educational sessions will be entered into REDCap by trained research assistants. These data are backed up continuously and protected by the University of Utah computer security systems.

CGM data are collected from Clarity [19] reports for 14 days at each time point and are stored in REDCap. The CDCESs access Clarity reports at each Share plus intervention session to provide dyadic coaching.

HbA_1c_ data are being collected using HbA_1c_ test kits, which require one drop of blood (similar to checking a glucose level) [53]. Participants mail the HbA_1c_ kits directly to the laboratory, which is traced with a tracking number to ensure privacy. This process will allow the study team to link the data to the participant once the HbA_1c_ data have been processed.

The risk of harm to participants, persons with diabetes, and their CPs is minimal. However, we have protocols in place if a participant is harmed. These protocols include reporting events to the institutional review boards and our data safety monitoring board in accordance with institutional and federal policies.

## Results

This study was funded in February 2023. We began enrolling participants on November 11, 2023. To date, 24 participants have been enrolled in this study. We expect to conclude this study in March 2026 and expect to disseminate results in March 2026.

## Discussion

### Expected Findings

The expected outcome is that the Share plus intervention will have a clinically significant intervention signal (increased TIR and lower diabetes distress) indicating readiness for a fully powered trial. The Share plus intervention examines dyadic support for using CGM with data sharing in older adults with T1D. The clinical promise of CP data sharing to improve diabetes management and glycemic levels, and thus, reduce complications, will be greatly enhanced through an intervention that removes barriers and strengthens dyadic communication and support for the growing number of older adults with diabetes.

### Conclusions

In this randomized controlled trial, we will evaluate feasibility and acceptability. By leveraging the full potential of technology and CP interventions, we may optimize the support that CPs can provide for effective glucose management in older adults with T1D.

## Abbreviations

ADCES7: Association of Diabetes Care and Education Specialists Educational Curriculum
CDCES: Certified Diabetes Care and Education Specialist
CGM: continuous glucose monitor
CP: care partner
DSME: diabetes self-management education
HbA_1c_: hemoglobin A1c
HIPAA: Health Insurance Portability and Accountability Act
QoL: quality of life
REDCap: Research Electronic Data Capture
SPIRIT: Standard Protocol Items: Recommendations for Interventional Trials
T1D: type 1 diabetes
TIR: time-in-range
